# Current and Emerging Topical Scar Mitigation Therapies for Craniofacial Burn Wound Healing

**DOI:** 10.3389/fphys.2020.00916

**Published:** 2020-07-29

**Authors:** Sun Hyung Kwon, Janos A. Barrera, Chikage Noishiki, Kellen Chen, Dominic Henn, Clifford C. Sheckter, Geoffrey C. Gurtner

**Affiliations:** Department of Surgery, Stanford University School of Medicine, Stanford, CA, United States

**Keywords:** burn injury, drug therapy, burn physiology, wound healing, facial burn

## Abstract

Burn injury in the craniofacial region causes significant health and psychosocial consequences and presents unique reconstructive challenges. Healing of severely burned skin and underlying soft tissue is a dynamic process involving many pathophysiological factors, often leading to devastating outcomes such as the formation of hypertrophic scars and debilitating contractures. There are limited treatment options currently used for post-burn scar mitigation but recent advances in our knowledge of the cellular and molecular wound and scar pathophysiology have allowed for development of new treatment concepts. Clinical effectiveness of these experimental therapies is currently being evaluated. In this review, we discuss current topical therapies for craniofacial burn injuries and emerging new therapeutic concepts that are highly translational.

## Introduction

Burn injuries represent one of the leading causes of accidental death worldwide. In the United States alone, burn incidents that demand medical treatment exceed more than 400,000 cases each year, ∼40,000 of which require hospitalization and intensive post-burn care (Berman et al., 2008; Murray et al., 2012; Sethi et al., 2014; Aba, 2016; Zhu et al., 2016). Over 50% of burn injuries involve the head and neck region and deep second and third-degree burns in this region often result in hypertrophic scar (HTS) formation leading to an assortment of debilitating effects both functionally and cosmetically (Sethi et al., 2014; Aba, 2016). Facial burns are particularly devastating to the affected patient who experiences psychological stress during acute treatment and subsequent lower quality of life when scars manifests. Reconstruction of burned skin, soft tissues, and other structures may require a combination of skin grafts, flaps, and tissue expansion techniques used to restore the functional and esthetic units of the craniofacial region. These patients may require long hospitalizations and multiple reconstructive procedures to prevent abnormal scar formation, including HTS and/or contracture (Ashab Yamin et al., 2015; Aba, 2016; Chua et al., 2016).

Despite modern advances in surgical techniques and biomedical technologies, management of facial burns remains challenging and post-burn scar formation may be inevitable. The approach to treating a burn wound begins with accurately diagnosing the depth and size of the burn, which is of critical importance for deciding the timing and approach to management. Superficial (first-degree) burns heal without the need for surgery and typically do not lead to adverse scar formation or hyperpigmentation as the entire dermis is preserved. Superficial partial thickness (second-degree) burns typically heal spontaneously within days to weeks and may result in scarring. Deep partial-thickness burns and full thickness (third-degree) burns usually require excision with reconstruction (often skin grafting) and are more prone to developing HTS. It is generally accepted that any burn that is not expected to heal on its own within 3 weeks should undergo excision and grafting as early as is feasible. This is due to the increasing risk of HTS that doubles from 20 to 40% between 2 and 3 weeks, as demonstrated by Cubison et al. (2006) in a study of pediatric scald burns. Even with precise operative technique, proper excision and grafting can still result in undesirable HTS that may not be cosmetically acceptable (Berman et al., 2008; Ashab Yamin et al., 2015; Chua et al., 2016; Zhu et al., 2016). Preventive therapies to reduce the formation of widespread HTS is fundamental to burn care and can significantly alter the outcome depending on the efficacy of treatment, timing of treatment, and duration of treatment. Silicone sheets, tapes, and gel formulations physically cover the healing area and are widely used as a general approach to scar mitigation (Meier and Nanney, 2006; Bleasdale et al., 2015). Over the past decade, a variety of advanced burn dressings have been introduced, many of them containing silver compounds for their long-standing antimicrobial effects. Other topical antibiotic agents and therapeutic occlusive or exposure dressings are all commonly used to facilitate healing and to prevent scar formation, although these non-molecularly targeted therapies have resulted in variable clinical outcomes (Leon-Villapalos et al., 2008; Block et al., 2015).

Although exuberant fibro-proliferation in the burn area starts as part of the normal wound healing, variations in cellular responses can lead to excessive scar-producing processes (Gurtner et al., 2008; Penn et al., 2012). Recent advances in our understanding of the cellular events and molecular signaling pathways underlying fibrotic scar development have led us to pursue investigational approaches to specifically targeted therapies. Rational manipulation of specific biological targets may hold promise for more effective and successful outcomes toward post-burn scar mitigation. Therapeutic agents involving growth factors, cytokine and other immunomodulators combined with new drug delivery technologies are being evaluated for their scar-improving properties (Asadullah et al., 2003; Rd Mag, 2011; So et al., 2011). More recently, cellular mechanotransduction modulators that can be applied topically have emerged as novel pharmacological agents that can accelerate wound healing while decreasing fibrotic scar formation (Gurtner et al., 2011; Wong et al., 2011b; Ma et al., 2018). In this review, we discuss current therapeutic modalities used for craniofacial burn wound and scar management and emerging experimental therapies that exhibit strong potential for clinical application.

## Overview of Craniofacial Burn Wound Healing

Burn injuries can vary widely in severity, ranging from local tissue damage to a complex systemic response which can quickly become life threatening. The complete local and systemic pathophysiology of burn injury has been reviewed in detail elsewhere and is beyond the scope of this review (Jewo and Fadeyibi, 2015; Kaddoura et al., 2017). Here we provide an overview of the localized effects of burns and burn wound healing in the craniofacial setting.

**FIGURE 1:**
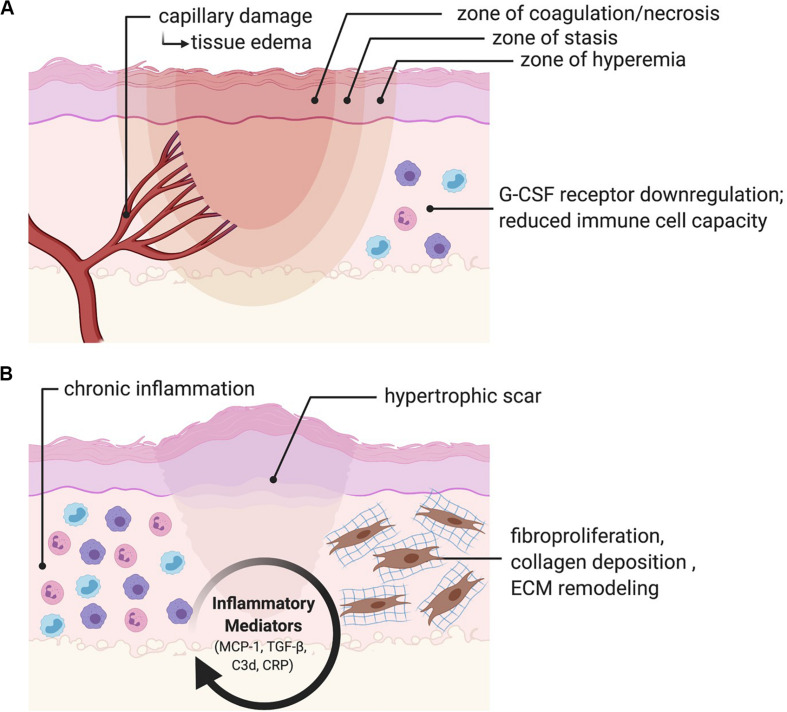
Burn pathophysiology. **(A)** Burn wounds are histologically characterized by a zone of coagulation/necrosis, a zone of stasis, and a zone of hyperemia. Microvascular damage results in a biphasic rise in fluid egress and localized edema. Immune function is depressed through disruption of the skin barrier, attenuated expression of granulocyte-colony stimulating factor (G-CSF) receptors, and a general reduction in immune cell capacity. **(B)** Persistent activation of inflammatory mediators including monocyte chemoattractant protein-1 (MCP-1), transforming growth factor-beta (TGF-β), complement component 3 (C3d) and C-reactive protein (CRP) results in chronic inflammation, fibroproliferation, collagen deposition and ECM remodeling, ultimately resulting in fibrosis and scarring.

At a molecular and cellular level, burn wounds are characterized by a surge in reactive oxygen species (ROS) production leading to oxidative stress, inflammation, and coagulative tissue necrosis (Ipaktchi et al., 2006; Parihar et al., 2008; van de Goot et al., 2009). Hypoxia can be measured at the burn wound margin at 48 h and peaks at 3 days post-burn, resulting in upregulation of hypoxia-inducible factor-1 alpha (HIF-1α) that promotes endothelial progenitor cell recruitment and neovascularization through its downstream effectors, including vascular endothelial growth factor (VEGF) and stromal cell-derived factor 1 (SDF1) (Gill et al., 2001; Fox et al., 2008; Xing et al., 2011). In superficial burns, skin protects the underlying tissues from heat propagation. However, these areas can be damaged depending on the severity of injury. Histologically, burn wounds are characterized by three zones (Figure 1):

1. Zone of coagulation/necrosis: this zone is characterized by complete vascular destruction and irreversible tissue loss.
2. Zone of stasis: this zone sustains a moderate degree of damage with some vascular damage leading to compromised tissue perfusion. This zone shows variable outcomes and may eventually recover or progress to necrosis.
3. Zone of hyperemia: this zone is characterized by vasodilation resulting in increased blood flow and hyperemia and will recover without any fibrosis or scarring.

In addition to direct tissue damage, burn injuries also result in microvascular fluid egress, which causes a sharp increase in localized edema that occurs within the first hour following injury (Arturson, 1961; Leape, 1972; Demling et al., 1978). Activation of the nitric oxide (NO) synthesis pathway has been found to significantly reduce burn edema, suggesting an important regulatory role in this process (Lindblom et al., 2000). A second, more gradual increase in edema peaks at 12–24 h post burn (Demling et al., 1978). The relative amount of edema seen in this second phase is dependent on whether or not fluid resuscitation is provided. When no fluid is administered, edema remains more self-limited (Lund et al., 1988; Onarheim et al., 1989). Immunologically, burn injuries cause a notable reduction in cellular immunity, which is attributed to attenuated expression of bone marrow Granulocyte Colony Stimulating Factor (G-CSF) receptors (Shoup et al., 1998). Larger burns are also correlated with the systemic release of inflammatory cytokines, including interleukin 6 (IL-6), IL-8, IL-10, and monocyte chemoattractant protein 1 (MCP-1) (Bergquist et al., 2019). In a study of 38 patients with major burns, Hiroshi et al. demonstrated that these four systemic inflammatory markers correlated with Sequential Organ Failure Assessment (SOFA) scores, while IL-6, IL-8, and IL-10 correlated with 28-day mortality (Matsuura et al., 2019). Finally, disruption of the skin barrier increases the susceptibility of the patient to infections caused by bacteria, fungus, and viruses. This susceptibility to infection is attributed to reduced effectiveness of all immune system components (Kaddoura et al., 2017).

Beyond the acute injury phase, burns are characterized by chronic activation of transforming growth factor-beta (TGF-β) and MCP-1, resulting in inflammation, fibroblast proliferation, collagen synthesis and deposition, and remodeling of the new extracellular matrix (ECM) (Roberts et al., 1986; Shallo et al., 2003; Penn et al., 2012). Persistent complement system activation, mediated by C3d and C-reactive protein (CRP), may also play an important role in promoting chronic inflammation in the burn wound (van de Goot et al., 2009). Fibrosis and scarring are the end result, and account for a significant amount of disability in burn survivors (Sheridan and Tompkins, 2004; Aarabi et al., 2007). Scar tissue is characterized by a highly vascular structure containing inflammatory cells and fibroblasts within a disorganized ECM (Singer and Clark, 1999). As a result, native tissue is replaced by a non-functional mass of tissue. In addition to its inferior mechanical properties as compared to regular uninjured skin (Corr and Hart, 2013), prolific fibrosis can result in HTS or keloid formation. HTS following burn injury is common in the craniofacial region and has been reported at 12–17% (Deitch et al., 1983), though there is a paucity of comprehensive clinical studies addressing this issue. While HTS only grows within the borders of the scar itself and tends to regress over time, keloids are characterized by continuous growth that extends beyond the borders of the wound and commonly recur following excision (desJardins-Park et al., 2019). Keloid formation occurs in individuals of all races (except albinos), although dark-skinned individuals carry a risk 15 times greater than lighter skinned individuals, or 6–16% (Gauglitz et al., 2011). In addition to these important functional considerations, HTSs and keloids can be unsightly, itchy, and painful. Furthermore, they can have a significant psychological impact on the patient. This is particularly important to consider in the facial area, as a person’s face is typically the main point of focus during social interactions (Sainsbury, 2009). Facial burns can disrupt a person’s body image and constitute a life-altering event. Treatment options for chronic hypertrophic scars and keloids include corticosteroid injections, chemotherapeutic injections, laser treatments, or surgery including scar release and skin resurfacing.

**FIGURE 2:**
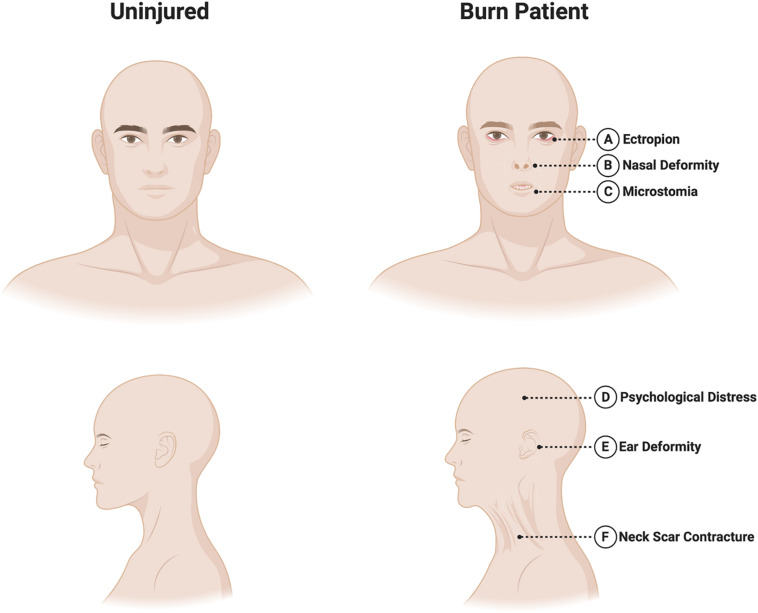
Sequelae of craniofacial burns. Burns in the craniofacial area requires special attention to the following areas. **(A)** Eyes/eyelids: eyelid burns can cause lid malposition and cicatricial ectropion or entropion, potentially leading to corneal ulceration or blindness. **(B)** Nose*:* the relatively vulnerable nasal blood supply, as well as potential damage to nearby vessels in the context of burn injury can make nasal reconstruction particularly challenging. **(C)** Mouth: perioral scarring can result in microstomia and difficulties with oral competence and speech. **(D)** Psychosocial health: the face plays a critical role in determining body-image. Patients can suffer psychological distress following burn injury including acute and post-traumatic stress disorders and depression. **(E)** Ears: the auricular cartilage lies directly beneath the skin and is vulnerable to infection if not protected. Severe deformities may require staged reconstructive surgery. **(F)** Neck: scar contractures of the neck can reduce neck mobility and interfere with mastication, phonation and breathing.

The craniofacial region contains a rich anatomy with many unique and specialized structures that deserve special consideration in the context of burn injury. The robust vascular supply of the head and neck allows for many areas to recover quite well from superficial partial thickness burns. However, deeper injuries to functionally and esthetically important areas can result in significant functional and cosmetic sequelae for patients. Several key anatomical areas are discussed below, and are illustrated in Figure 2.

### Eyes/Eyelids

Burn injuries of the eyelids can cause lid malposition resulting in cicatricial ectropion or entropion, potentially leading to long-term problems with vision including corneal ulceration and even blindness (Liu et al., 2011). All three lamellae of the eyelid may require reconstruction in the acute setting, although injuries are usually limited to skin grafting of the anterior lamella. In patients with large TBSA% burns that develop conjunctivitis and exposure keratopathy, a reversible tarsorrhaphy may be considered when timely lid excision and reconstruction is not possible (Klein et al., 2008).

### Ears

The auricle deserves special consideration given its cartilaginous structure which lies directly underneath the skin. The cartilage is therefore at risk of chondritis following full thickness burn injury (Kraenzlin et al., 2018). Topical antimicrobials capable of penetrating cartilage, such as sulfamylon, are critical in this setting. Burn scars can result in ear deformities requiring excision and reconstructive procedure ranging from skin grafts to total auricular reconstruction using cartilage grafts and free flaps (Ibrahim and Salem, 2008).

### Nose

Nasal burns can cause facial disfigurement that can negatively impact patients’ psychosocial health (Bouguila et al., 2017). These injuries can be particularly challenging to treat given that adjacent facial tissues may also be damaged. This can limit reconstructive options, which typically depend heavily on local flaps (Prousskaia et al., 2015). Furthermore, the pattern of blood supply makes it especially vulnerable to the effects of injury, ischemia and secondary infection. Despite these challenges, nasal tissue is rich with adnexal structures and sebaceous units, allowing rapid re-epithelialization. Reconstructive options include skin or composite autografts, allografts, dermal substitutes, locoregional flaps, and/or free flaps.

### Mouth

Burn scarring of the perioral region can result in purse-stringing of the oral aperture, microstomia, drooling, shortening of the columella, and cicatricial ectropion of both the upper and lower lips (Neale et al., 1985). These changes may affect speaking, eating, drinking, and maintaining oral hygiene. Surgical treatment options include scar release with skin grafting, Z-plasty, and/or commissuroplasty.

### Neck

Scarring in the neck region can result in contractures that limit neck mobility, mastication, phonation, or breathing, and may cause neck pain and esthetic concerns (Akita et al., 2017). Treatment approaches include scar release with skin grafting, regenerative dermal matrix templates, or locoregional or distant flaps to bring in well-vascularized tissue. Recent innovations in reconstructive surgery have led to the development of “superthin” free perforator flaps, such as the supercharged occipito-cervico-pectoral (OCP) flap, which can provide excellent recontouring of the neck and face following scar excision (Vinh et al., 2015).

### Oral Mucosa

In contrast to the scarring phenotype seen on the exterior head and neck, the oral cavity is characterized by a remarkable ability to heal with minimal scarring (Wong et al., 2009). With the exception of massive ingestion of strong acids or bases and electrical burns of the mouth, most mucosal tissues heal rapidly and require little treatment (Baruchin et al., 1991).

Given the remarkable advances in acute burn resuscitation and care that have evolved over the past century, burn injuries are becoming an increasingly survivable injury. As a result, preventing, minimizing or correcting the long-term sequelae of burn scars is becoming increasingly important for improving the quality of life of patients. In the following sections we will review the history and advancement of burn care, discuss current therapies for craniofacial burn treatment and discuss new and emerging therapies to reduce post-burn scarring.

## History and Advancement of Burn Wound Treatment

Unfortunately, burn injuries are also as old as our own history. The ability to create and manipulate fire is one of the quintessential characteristics of mankind dating back millennia. There is a rich history of our understanding of burns and approach to burn care, found in texts dating back to ancient Egypt (Artz, 1970). In parallel with our evolving understanding of burn depth and pathophysiology, burn treatments have evolved significantly over the years. In the ancient Egyption Ebers papyrus, a 5-day regimen of cattle dung, bees wax, ram’s horn, and barley porridge soaked in resin was described for topical burn treatment (Liu et al., 2017). Chinese and Japanese texts described the use of tea leaves for treating burns in the 5th–6th centuries B.C. (Artz, 1970). Around 400 B.C., the Greek physician Hippocrates described the use of porcine skin mixed with a resin of bitumen, spread on a piece of warmed cloth, which was alternated with warm vinegar-soaked dressings and oak-bark tanning solutions (Artz, 1970). Celsus of ancient Rome described treating burns with honey and bran, followed by cork and ashes.

Ambroise Paré was likely the first to describe early burn wound excision in the mid-16th century (Liu et al., 2017). Later, in the 1940s, several surgeons including Young McCorkle and Silvani, and Saltonstall and Lee reported extensive experience with full thickness burn excision (Young, 1942; Saltonstall and Lee, 1944; McCorkle and Silvani, 1945). In the late 1950s, Jackson et al. reported a series of trials testing immediate fascial excision and grafting of small burn areas, eventually covering burns up to 65% TBSA with skin grafts (Jackson et al., 1960). Janzekovic is credited for introducing tangential excision which allowed early burn excision to gain widespread acceptance (Janzekovic, 1970, 1972; Janžekovič, 2008). She reported a large series of 2615 patients with deep partial thickness burns who underwent tangential excision with immediate grafting 3–5 days after injury (Janzekovic, 1970). A retrospective review by Tompkins et al. in 1986 of burn patients at Massachusetts General Hospital found an astounding improvement in mortality across all patients admitted for burn injury, from 24% in 1974 to 7% for 1979–1984, following the institutionalized practice of early excision and grafting (Tompkins et al., 1986). The benefits of early excision and grafting, including reduced hospital stay and reduced mortality, have been replicated in more recent clinical studies and meta-analyses (Tompkins et al., 1988; Herndon et al., 1989; Ong et al., 2006).

Following the Cocoanut Grove nightclub fire in Boston, MA in 1942, which killed and injured hundreds of patients, doctors noted that larger burns would result in hypovolemic shock without fluid resuscitation (Cartotto, 2009). Prior to this, Underhill had proposed using blood hemoglobin percentage as an index of resuscitation, and felt that preventing hemoconcentration in the acute phase following burn injury was necessary to maximize survival (Underhill, 1930). In 1944, Lund and Browder developed diagrams still used today to facilitate acute TBSA measurement (Lund and Browder, 1944). Several formulas for guiding fluid resuscitation were proposed over the following years, with the most famous and widely used today being the Parkland formula introduced by Baxter and Shires in 1968, which recommends 4 mL of lactated ringer’s (LR)/kg/% TBSA burned during the first 24 h of resuscitation, where the first half the resuscitative volume is given within the first 8 h and the second half is administered over the next 16 h (Baxter and Shires, 1968).

Fluid resuscitation, early excision and grafting, and infection control have led to significant improvements in burn morbidity and mortality. In the following sections we explore in detail modern methods of craniofacial burn wound care, as well as new and emerging therapies targeting post-burn scar mitigation.

## Current Topical Therapies for Craniofacial Burn Wound Management

**FIGURE 3:**
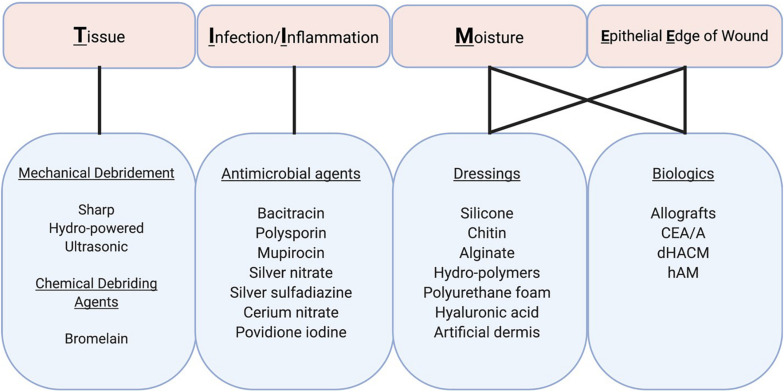
Topical therapies for craniofacial burns. Treatment and prognosis are largely determined by the size and location of partial and full thickness burns. TIME (Tissue, Infection/Inflammation, Moisture imbalance, and Epithelial Edge of wound) describes four aspects of wound bed preparation that need to be systematically addressed for wound healing to take place. These components are inter-related although the relative importance of each in a particular wound varies. Therapeutic actions can address several of these components, albeit with variable degrees of effectiveness. CEA/A, cultured epithelial allografts/autografts; dHACM, Dehydrated human amnion/chorion membrane; hAM, human amnion membrane.

Most craniofacial burns spontaneously heal and do not require surgery given the head and neck are well vascularized. Non-operative therapies for post-burn scarring are primarily aimed at reducing the risk of wound site infection and facilitating the debridement of necrotic tissue. The TIME (Tissue, Inflammation/infection, Moisture imbalance, Epithelial edge advancement) concept represents a systematic approach for assessing and treating wounds, and provides a useful reference for categorizing topical burn therapies (Figure 3; Harries et al., 2016). The current standard of care for acute burns involves topical treatment with antimicrobial preparations. Once the wound epithelializes, topical antimicrobials should be discontinued and various intervention options could be employed to prevent HTS, including scar massage, compression garments, and silicone sheeting. Sun avoidance/protection is also critical as newly healed skin is fragile and prone to sun-burning and hyperpigmentation. When HTS is starting to form, cryotherapy, corticosteroid injections, and surgical interventions are utilized with variable outcomes.

### Antimicrobial Agents

To prevent infection, silver preparations such as silver sulfadiazine (SSD) and silver-containing dressings are commonly used for the treatment of second and third-degree burns. However, these agents are discouraged in the face due to their adverse effects on mucosal membranes of the eye, nose, and mouth. Topical ointments such as bacitracin, polymyxin, and mupirocin are used instead (Leon-Villapalos et al., 2008; Maillard and Hartemann, 2013). Silver nitrate is another topical therapy, however, studies have reported that silver nitrate directly reduces fibroblast proliferation, and it is therefore not recommended for prolonged or excessive use (Maillard and Hartemann, 2013). For the neck, SSD is often used in combination with cerium nitrate, another topical antiseptic agent that augments its antimicrobial effects (Garner and Heppell, 2005; Leon-Villapalos et al., 2008). Topical silver therapies have been associated with silver deposition in the liver and kidney, and therefore should be used with caution in pediatric and elderly patients, as well as in large surface area burns (Orsini and Plastic Surgery Educational Foundation Technology Assessment Committee, 2006; Leon-Villapalos et al., 2008; Friedstat and Klein, 2009). Additionally, use of silver-based therapies can result in leukocytopenia, so white blood cell count monitoring should be considered for patients requiring prolonged therapy (Choban and Marshall, 1987). Antimicrobials should be discontinued once epithelialization has completed so that anti-scar therapy can begin.

### Debriding Agents

Early debridement allowing for healthy tissue regrowth is the cornerstone of burn wound care and is vital to the overall outcome of burn patients. The practice of excising burn eschar in the facial area, however, remains controversial, given its robust blood supply which is favorable to spontaneous healing within an acceptable period. For debridement, surgical excision and/or proteolytic enzymes that digest necrotic tissues can be used. Surgical excision may be performed sharply using a Weck, Goulian, or Watson blade. Alternatively, mechanical debridement may also be performed using hydro-powered devices such as the Versajet (Smith and Nephew, London, United Kingdom), or ultrasonic debridement devices. Bromelain-based agents are most used for enzymatic debridement, however, this method can slow healing time and cause significant pain. For these reasons, use of enzymatic agents has been generally avoided for facial burns thus far.

### Growth Factors and Cytokines

Growth factors and cytokines play a key role in wound healing, including the promotion of proliferation and migration of various cell types, recruitment of circulating inflammatory and progenitor cells, and stimulation of angiogenesis and ECM production (Ching et al., 2011). Topical treatment with growth factors and cytokines has demonstrated positive results in preclinical and clinical trials, especially in the setting of partial thickness burns. Growth factors with positive human clinical data include fibroblast growth factor (bFGF), recombinant human epidermal growth factor (EGF), and granulocyte-macrophage colony-stimulating factor (rhGM-CSF) (Fu et al., 1998; Zhang et al., 2009; Guo et al., 2010). Other growth factors such as platelet-derived growth factor (PDGF) and vascular endothelial growth factor (VEGF) have demonstrated positive effects on burn healing in preclinical models but have yet to be demonstrated to be efficacious in human patients (Ching et al., 2011). A detailed discussion of transforming growth factor-beta (TGF-β), interleukins, and novel mechanomodulatory agents is provided later in this review.

### Therapeutic Wound Dressings

Burn wound dressings are made from a variety of materials including silicone, chitin, alginate, hydro-polymers, and polyurethane foam. Dressings containing therapeutic agents such as aforementioned silver compounds are also used. Use of hyaluronic acid-based wound dressings has been reported to be safe and effective for partial-thickness facial burns. Silicone sheet dressings have been shown to soften post-burn scars, prevent contracture and increase skin and joint mobility. These effects are attributed to greater tissue hydration, which results in improved scar texture and color (Leon-Villapalos et al., 2008; Bleasdale et al., 2015).

### Biologic and Biosynthetic Skin Substitutes

Dermal substitutes are bio-matrices that provide a protective layer over open wounds, protecting against infection, reducing pain and stimulating a healing response to allow for eventual skin grafting (Shahrokhi et al., 2014). As in other parts of the body, these skin substitutes constitute an important mechanism for covering deep burn wounds in the face, typically in the setting of large TBSA% burn wounds or in cases where there are limited donor sites available for autografting. The first product introduced into the market was Integra (Integra^®^ Life Science Corporation, Plainsboro, NJ, United States). Originally developed by Harvard and MIT scientists, Integra^®^ is an artificial acellular bilayer composed of a bovine collagen type I and chondroitin-6-sulfate dermis, with a semipermeable silicone membrane “epidermis.” The silicone membrane is left in place for at least 2 weeks, allowing for sufficient neovascularization to take place to allow for skin grafting over the dermal substitute (Davison-Kotler et al., 2018).

Acellular dermal matrices (ADMs) benefit from providing natural dermal porosities to support tissue regeneration and neovascularization while providing wound protection (Chua et al., 2016).

For example, Alloderm^®^ (Allergan, Dublin, Ireland) is a human cadaveric allograft which is chemically decellularized using mild non-denaturing detergents, while the collagen matrix is preserved. Bioengineered allograft materials can also contain cellular components, such as Apligraf^®^ (Organogenesis, Inc., Canton, MA, United States), a cellular bilayer substitute containing bovine collagen type I dermis, as well as allogenic neonatal keratinocyes and fibroblasts (Davison-Kotler et al., 2018). A recent clinical trial found that Apligraf placed over meshed autografted burn wounds resulted in improved pigmentation, tissue pliability, vascularization and scar appearance, as opposed to regular meshed autografted wounds (Waymack et al., 2000). Although the host immune response to allograft precludes its use as a permanent skin replacement, achieving temporary wound coverage with allograft stimulates the release of a variety of bioactive substances that accelerate wound closure (Katz and Taichman, 1994). Clinical trials have shown that skin allografts can accelerate the healing of burn wounds as well as chronic wounds in the lower extremity (Nuñez-Gutiérrez et al., 1996; Gurtner et al., 2020). It has been shown that the viability of skin allografts correlates with take rate (Bravo et al., 2000; Castagnoli et al., 2003), thus effective techniques for preservation of skin grafts are critical to maintain high quality grafts. In light of the high demand for allograft skin especially for the treatment of major burns, allograft preservation in skin banks has become an established practice. Cryopreservation is the preferred method of cadaveric skin preservation, since it better maintains the physicochemical properties and viability of fresh human skin compared to glycerol (Aggarwal et al., 1985; Cinamon et al., 1993; Pirnay et al., 2012). Recently developed human cryopreserved meshed split-thickness skin allografts such as TheraSkin^®^ (Misonix, Farmingdale, NY, United States) have shown promising results in the treatment of chronic wounds in clinical trials (DiDomenico et al., 2011; Gurtner et al., 2020) and demonstrated regenerative properties in pre-clinical studies (Henn et al., 2020), suggesting a potential future therapeutic role in burn care as well.

Human amnion/chorion can be a source of cellular and acellular biologic scaffolds. Dehydrated human amnion/chorion membrane (dHACM) allografts such as EpiBurn or EpiFix, can be used to protect the wound while promoting vascular angiogenesis and healing (Reilly et al., 2017). Similarly, decellularized human amnion membrane (hAM) contains a favorable immunogenic profile, has antimicrobial and anti-fibrotic properties (Robson and Krizek, 1973; Maral et al., 1999), and can reduce the frequency of dressing changes, which is particularly useful in pediatric patients (Branski et al., 2008). Over the past 20 years, there has been an increasing body of literature describing hAM processing methods and clinical use (Herndon and Branski, 2017). Challenges related to its relative fragility and higher cost have thus far limited widespread clinical use (Quinby et al., 1982).

Finally, xenografts offer an alternative method for obtaining wound coverage with the added benefit of improved cost and availability, as compared to allograft materials. Compared to human ADM, xenograft ADM has demonstrated strong protein homology and equal biocompatibility (Ge et al., 2009). Examples of xenografts include: SurgiMend (TEI Biosciences, Boston, MA, United States) acellular bovine dermis; Strattice (LifeCell, Branchburn, NJ, United States) acellular porcine dermis; and Permacol (Covidien, Dublin, Ireland) acellular porcine dermis.

## New and Emerging Therapies for Post-Burn Scar Mitigation

While various physical therapies and operative procedures can facilitate burn wound healing and minimize post-burn scars, use of these treatment modalities is not always suitable for craniofacial burns. Topically applied pharmacological agents are also effective treatments, especially during the acute phase, but choices are limited. Antimicrobial and enzymatic agents have been used primarily to prevent infection and promote debridement of necrotic tissues. Several vitamin preparations and plant-based remedies are also available. However, many topical therapies lack concrete scientific evidence and results have been variable. As our knowledge of the mechanistic basis of wound healing and scar development improves, approaches to develop molecularly targeted therapies have shown promise (Whittam et al., 2019). These therapies can be widely applied for the treatment of burns and other forms of deep cutaneous injuries. The importance of developing more effective and safer therapies is critical to facial burn scars as successful outcomes can remarkably change the quality of life of patients. Improved understanding of these treatment modalities will also lead to the opportunity for developing effective therapies specific to craniofacial burns.

### Transforming Growth Factor-β (TGF-β)

TGF-β expression is involved in almost all stages of the wound healing process, including inflammation, angiogenesis, re-epithelialization, ECM synthesis, and wound remodeling. Many studies have established the pivotal role of this growth factor in myofibroblast differentiation and subsequent scar formation across a range of fibrotic diseases (Meier and Nanney, 2006; So et al., 2011; Pakyari et al., 2013). Studies exploring the scar-less and the regenerative ability of the human fetus have pointed toward the possibility that different isoforms of TGF-β could contribute to different stages of healing. While TGF-β1 and TGF-β2 are highly expressed in adults, TGF-β3 is the dominant isoform in fetal wound healing, leading many researchers to identify either TGF-β3 as a therapeutic target to upregulate or TGF-β1/β2 as targets to suppress for scar-less wound healing (Meier and Nanney, 2006; So et al., 2011; Pakyari et al., 2013).

Despite strong pre-clinical outcomes, current TGF-β clinical trials have had disappointing results. Juvista (Renovo, United Kingdom), a recently-developed recombinant TGF- β3 product, had shown promise in early phase efficacy trials but failed to meet primary endpoints in a Phase III trial (Rd Mag, 2011; So et al., 2011). Juvidex (mannose-6 phosphate, Renovo, United Kingdom), an inhibitor of TGF-β1/TGF-β2, also failed to meet the main study goals in a Phase II trial (Meier and Nanney, 2006). Other researchers have also explored the clinical potential for a recombinant human antibody to neutralize TGF-β1 for systemic sclerosis, but their clinical studies did not prove any difference in efficacy from control during their Phase I/II trials (Meier and Nanney, 2006). TGF-β therapy could potentially have confounding results due to its dual importance in both normal wound healing but also in excessive fibro-proliferation during HTS formation. TGF-β receptors are upregulated during fibrosis, and while blocking TGF-β expression seems to prevent fibrosis, it can also lead to chronic, non-healing wounds (Gabriel, 2009; Pakyari et al., 2013). Albeit, TGF-β continues to be an attractive pharmacological target for a wide variety of fibrotic diseases, and newer strategies modulating TGF- β pathophysiology awaits further investigation.

### Interleukins (IL)

Neutrophils and macrophages, two of the major cell types during the inflammatory phase of burn injury, secrete cytokines such as interleukins (IL) and tumor necrosis factor β (TNF-β) (Asadullah et al., 2003). IL-10 has been shown to regulate inflammatory cell function during wound healing by sequestering pro-inflammatory IL-6 or IL-8 and by regulating the T cell cytokine production, leading some researchers to explore the therapeutic effect of administering IL-10 to the wound bed during early wound healing (Asadullah et al., 2003). These studies demonstrated the ability of IL-10 to potentially improve scar healing in human patients during Phase I/II trials administering Prevascar (Renovo, United Kingdom), a recombinant human IL-10 (rhIL-10) product applied intradermally (Meier and Nanney, 2006). Treatment with rhIL-10 has also been explored in several other clinical trials to combat various inflammatory diseases (Asadullah et al., 2003). Other wound cytokines such as IL-2 may also play a role in altering the properties of wound healing presumably by contributing to the resolution of inflammation. Although studies have shown that local treatment of wounds with IL-2 can improve the strength of healed skin, clinical use of IL-2 faces major obstacles such as systemic toxicity due to narrow therapeutic window (Doersch et al., 2017).

### Mechanomodulatory Agents

Mechanical stress is an important component of wound healing and plays a pivotal role in facilitating pro-fibrotic events via cellular mechanisms that stimulate inflammatory pathways (Gurtner et al., 2008, 2011; Wong et al., 2011a, b; Januszyk et al., 2014; Ma et al., 2018). Over-activation of these pathways can lead to excessive fibro-proliferation. Physically reducing mechanical stress with new devices that can modulate local biomechanics, therefore, has gained a rapidly growing market for scar reduction of surgical wounds. Based on long-standing surgical principles used to minimize scar development, these polymer-based medical devices offload mechanical force to release tension imposed upon healing incisions (Gurtner et al., 2011; Januszyk et al., 2014). Incisions created at high-tension body locations such as the central chest, shoulders, knees, ankles, and/or the back are prone to forming HTS than other body sites. For example, abdominoplasty wounds can develop into wide scars due to their natural high-tension closure, and application of a stress-shielding device on these wounds have demonstrated clear efficacy in mitigating scar formation (Kwon et al., 2017). This technology successfully led polymer stress-shielding devices to the market, and numerous patients have seen benefits in the clinic.

**FIGURE 4:**
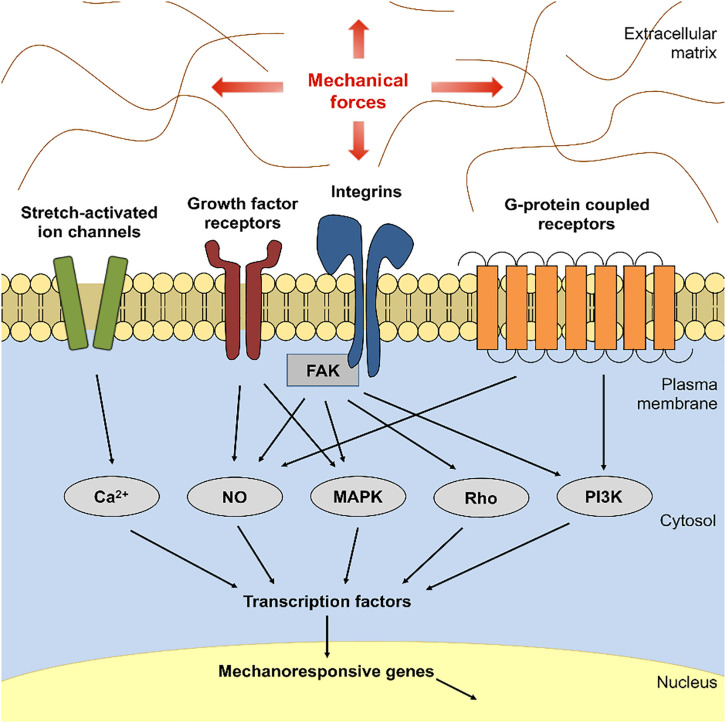
Focal Adhesion Kinase (FAK)-mediated mechanotransduction. Schematic demonstrates that mechanical forces activate Integrin-FAK, resulting in activation of several downstream effector proteins and transcriptional factors that mediate cellular mechanotransduction. Over-activation of these signals can result in hypertrophic scar formation upon cutaneous injury. NO, nitric oxide; FAK, focal adhesion kinase; MAPK, mitogen-activated protein kinase; PI3K, phosphoinositide 3-kinase.

Despite success on surgical incisions, polymer mechanomodulatory devices are difficult to use on large size excisional wounds, burn injuries, and wounds that formed in contoured body areas such as the facial area. Alternatively, non-invasive therapeutics that pharmacologically target key mechanotransduction pathways (cellular machinery that transduces mechanical stimuli to biochemical signals; Figure 4) have also received highlight in recent literature (Wong et al., 2011b; Kwon et al., 2017; Ma et al., 2018). Currently, a prototype of such therapeutic agents is at the early pre-clinical development stage with highly translational potential (Wong et al., 2011b; Kwon et al., 2017; Ma et al., 2018).

Therapeutic suppressors of mechanomodulatory proteins have been studied as anti-cancer agents for many decades. A non-receptor protein tyrosine kinase, focal adhesion kinase (FAK), is a key upstream mediator of the Integrin mechanotransduction pathway and an important inducer of cell adhesion, proliferation, migration, and angiogenesis (Parsons, 2003; Wong et al., 2011b; Ma et al., 2018). FAK is known to be deregulated in cancer and is thought to be a rational target to block tumor activities using pharmacological inhibitors (Parsons, 2003). Because emerging studies have shown that FAK transduces mechanical stress signals to stimulate the activation of the FAK–ERK–MCP-1 signaling pathway and is an important regulator of cancer-promoting pathways (Wong et al., 2011b; Ma et al., 2018), many pharmaceutical companies have taken effort to develop later generation FAK inhibitors that display improved pharmacodynamic and pharmacokinetic properties (Parsons, 2003; Infante et al., 2012).

In current literature, small molecule-mediated inhibition of the kinase activity of FAK has been successfully used to prevent mechanically-induced skin HTS formation (Wong et al., 2011b; Kwon et al., 2017; Ma et al., 2018). FAK inhibition significantly reduced scar-forming fibroblast migration, myofibroblast production of α-smooth muscle actin, and aberrant collagen deposition (Parsons, 2003; Wong et al., 2011b; Ma et al., 2018). FAK inhibitor therapy that can be safely delivered to wound sites would be a new and promising approach to wound and scar management. In most circumstances, cutaneous scar development is a localized event; therefore, this provides rationale and support to develop targeted FAK inhibitor delivery modalities in attenuating scar formation. Localized drug delivery has the advantage of circumventing systemic toxicity while maximizing bioavailability and local drug efficiency.

Medicated self-adhesive patches that release therapeutic molecules upon dermal contact may not be suitable for large size burn wounds, and other topical formulations in the form of topical cream, lotion, or ointment are easily subject to over- or under-dosing if application amount per treatment is not accurately measured. The latter is also problematic especially for compounds with potential adverse effects at the systemic level (Infante et al., 2012). Therefore, it is scientifically reasonable to devise a dermal delivery vehicle that can deliver topical FAK inhibitor to wounds and/or scars in a controlled manner. Biopolymers have strong advantages as drug delivery carriers because of their ability to carry bioactive compounds to target tissues and cells and to release compounds over a prolonged duration (Wong et al., 2010). Recent studies have used collagen-based bioscaffolds to develop biodegradable hydrogels that release FAK inhibitors in a regulated manner upon direct contact with the skin, which has proven pre-clinical efficacy in rodent wound models for HTS reduction (Wong et al., 2011a; Kwon et al., 2017; Ma et al., 2018). Development of optimal drug delivery systems and extensive safety studies on potential adverse effects of each agent will be imperative to successful development of pharmaceutical therapies to treat wounds and scar formation.

## Cell-Based Therapies

Cell-based therapies have emerged as an important component of regenerative medicine. Stem cells of various sources and cultured dermal cell devices such as epithelial sheets have shown merit when used as an alternative or in conjunction with standard skin grafting techniques for therapeutic application in severe burn injuries. The technology, however, are highly regulated by authorities globally and consistent concerns have been raised as to the biosafety and clinical efficacy of cell-based therapies (Li and Maitz, 2018).

Despite scientific concerns, many autologous and allogeneic cell products were developed over the past decades using cells of skin and non-skin origins in burn wound management (Li and Maitz, 2018). Cultured epithelial allografts (CEA) prepared in advance can be used for temporary coverage of acute burns as a bridge to eventual cultured epithelial autografting. A recent cost-utility analysis found that skin allograft resulted in greater quality-adjusted life years (QALY) compared with topical silver dressings at a higher cost and may be a considered a cost-effective treatment in certain settings for partial-thickness burn (Sheckter et al., 2020). CEAs are frequently used in combination with meshed skin grafts to facilitate wound closure (Katz and Taichman, 1994; Sheckter et al., 2014). Dermal fibroblast treatments are also effective as these cells produce important ECM proteins and growth factors and cytokines that work synergistically when used with a regenerative scaffold (Spiekstra et al., 2007; Li and Maitz, 2018). Keratinocyte stem cells are important for regulating epithelial stratification and regeneration of hair follicles and other skin appendages (Blanpain and Fuchs, 2006; Li and Maitz, 2018). These properties have potentiated the use of keratinocyte stem cells for burn wound management. More recently, research using undifferentiated stem cells and progenitor cells for burn wounds have shown progress (Li and Maitz, 2018). Studies have shown that mesenchymal stem cells administered both systemically and locally exhibited therapeutic effects promoting wound closure and tissue regeneration while reducing fibrosis and scar formation (Caplan and Dennis, 2006; Duscher et al., 2015; Li and Maitz, 2018). Research also demonstrated that human embryonic stem cells and induced pluripotent stem cells (iPSCs) have potential applications as temporary skin substitutes for burn patients waiting for autologous skin grafting. Use of human embryos are, however, associated with ethical issues and safety concerns related to iPSCs should be resolved to leverage the full potential of cell-based therapies.

## Clinical Evaluation of New Scar Mitigation Therapies

Traditional evaluation of post-burn scarring is primarily based on subjective measurements such as scar assessment scales. However, comparative success of new experimental therapies should also depend on objective assessments that use a variety of standardized techniques and devices. Detailed discussion of these tools is beyond the scope of this review and can be found elsewhere (Lee et al., 2016). Regardless of which biological process is targeted, new therapies should aim to improve burn scar functional and mechanical properties such as pliability, viscoelasticity, firmness, and perfusion in addition to visual improvements of healed skin. These properties can be measured using non-invasive dermatological tools used in the cosmetic industry, which can perform quantitative analyses of various scar parameters. As many of the new scar mitigation technologies are still in their developmental phase, clinical effectiveness of scar treatments should be determined by a combination of objective assessment tools to declare success over traditional therapies.

## Conclusion

Burn scarring and fibrosis are responsible for a significant burden on patients and healthcare systems across the globe. The sequelae of these injuries can be especially challenging to treat in the craniofacial area given its unique functions and its significant relevance for psychosocial health. Although the craniofacial area benefits from robust vascularity and healing, careful consideration must be given toward injuries in particularly sensitive areas, such as the eyelids, nose, ears, mouth, and neck, as these require special reconstructive considerations. As our understanding of wound healing and fibrosis evolves, new minimally invasive therapies may offer exciting alternatives to traditional tangential excision and skin grafting, potentially leading to improved outcomes for patients and significant cost savings. Research is ongoing to identify and translate promising new therapies into the clinical arena.

## Author Contributions

SK, JB, CN, KC, DH, CS, and GG wrote the manuscript. All authors contributed to the article and approved the submitted version.

## Conflict of Interest

The authors declare that the research was conducted in the absence of any commercial or financial relationships that could be construed as a potential conflict of interest.
